# Self-reported late effects and long-term follow-up care among 1889 long-term Norwegian Childhood, Adolescent, and Young Adult Cancer Survivors (the NOR-CAYACS study)

**DOI:** 10.1007/s00520-020-05790-6

**Published:** 2020-10-03

**Authors:** A. V. Mellblom, C. E. Kiserud, C. S. Rueegg, E. Ruud, J. H. Loge, S. D. Fosså, Hanne C. Lie

**Affiliations:** 1 Department of Behavioural Medicine, Institute of Basic Medical Sciences, Faculty of Medicine, University of Oslo, P.B. 1111, 0317 Oslo, Norway; 2 National Resource Centre for Late Effects after Cancer Treatment, Oslo University Hospital, Radiumhospitalet, Oslo, Norway; 3 Oslo Centre for Biostatistics and Epidemiology, Oslo University Hospital, Oslo, Norway; 4 Department of Pediatric Haematology and Oncology, Division for Paediatric and Adolescent Medicine, Oslo University Hospital, Oslo, Norway; 5 Institute of Clinical Medicine, Faculty of Medicine, University of Oslo, Oslo, Norway

**Keywords:** Late effects, Follow-up care, Childhood cancer survivors, Adolescent and young adult cancer survivors

## Abstract

**Purpose:**

The majority of childhood, adolescent, and young adult cancer survivors (CAYACS) are at risk of late effects but may not receive long-term follow-up care for these. Here, we investigated (1) self-reported late effects, (2) long-term follow-up care, and (3) factors associated with receiving follow-up care in a population-based sample of Norwegian long-term CAYACS.

**Methods:**

Survivors were identified by the Cancer Registry of Norway. All > 5-year survivors diagnosed between 1985 and 2009 with childhood cancer (CCS, 0–18 years old, excluding CNS), breast cancer (BC, stages I–III), colorectal cancer (CRC), leukemias (LEUK), non-Hodgkin lymphoma (NHL), or malignant melanoma (MM) at age 19–39 years were mailed a questionnaire (NOR-CAYACS study). Descriptive statistics and logistic regression models were used to analyze occurrence of late effects, long-term follow-up care for these, and associated factors.

**Results:**

Of 2104 responding survivors, 1889 were eligible for analyses. Of these, 68% were females, with a mean age of 43 years at survey, on average 17 years since diagnosis, and diagnosed with CCS (31%), BC (26%), CRC (8%), NHL (12%), LEUK (7%), and MM (16%). Overall, 61.5% reported the experience of at least one late effect, the most common being concentration/memory problems (28.1%) and fatigue (25.2%). Sixty-nine percent reported not having received long-term follow-up care focusing on late effects. Lower age at survey (*p* = 0.001), higher education (*p* = 0.012), and increasing number of late effects (*p* = < 0.001) were associated with increased likelihood of follow-up care in the multivariate model.

**Conclusions:**

The majority of survivors reported at least one late effect, but not receiving specific follow-up care for these. This indicates a need for structured models of long-term follow-up to ensure adequate access to care.

**Electronic supplementary material:**

The online version of this article (10.1007/s00520-020-05790-6) contains supplementary material, which is available to authorized users.

## Background

The growing population of childhood, adolescent, and young adult cancer survivors (CAYACS) is at risk of significant cumulative disease burden due to late effects of the cancer and/or the treatment [1–4]. Late effects can occur in any organ system, and include among others secondary cancers, cardiovascular diseases, hormonal dysfunctions, neurocognitive problems, fatigue, and psychological distress [1, 2, 5–11]. Compared with the general population, survivors of childhood and adolescent cancers have twice the burden of disease, with an average of seven or more adverse chronic health conditions by the age of 50 [10]. Occurrence of late effects in the adolescent and young adult cancer survivor population is less studied than in childhood cancer survivors, but the available literature suggests similar trends [12].

Risk-adapted, long-term or life-long, follow-up care is therefore recommended [13–18], although research documenting the effect of such care is currently scarce [18]. Recommendations and several guidelines for such care exist [19–21], but there is great variability in the availability, organization, content, and duration of such care both within and between countries, and especially for the population of long-term CAYACSs [17, 22]. Even when available, attendance rates tend to be low due to high rates of disengagement from or being lost to long-term follow-up among young cancer survivors [23–26].

Follow-up care is important not only for early detection and management of manifest late effects but also to educate the survivors about their risks of late effects and to motivate them for healthy lifestyle behaviors [1, 27, 28]. The lack of long-term follow-up care combined with survivors’ reported lack of knowledge of late effects [29–31] might hamper the survivors’ opportunities for self-management of their long-term health.

In countries with publicly funded, free-for-all health care, such as Norway, cancer survivors should have access to care for late effects, even in the absence of formal long-term follow-up care models. In Norway, cancer patients are followed up by the specialist healthcare service through standardized care programs for the first 5–10 years after treatment completion. Thereafter, they contact their assigned general practitioner for later health concerns. To what extent adult, long-term survivors of childhood, adolescent, and young adult cancers receive follow-up care for late effects in a publicly funded healthcare system without a formal model for such care is not well researched. However, Canadian data suggests, however, that, even with free access to care and the existence of survivorship clinics, most adult survivors of childhood cancers do not attend [26].

Moreover, there is also sparse knowledge of the occurrence of self-reported late effects in an unselected population of long-term CAYACS not engaged in survivorship care programs. In Norway, the entire population of cancer survivors can be identified and tracked through the Cancer Registry of Norway (CRN). Taking advantage of this opportunity, we launched the NOR-CAYACS study in 2015–2016, a national, population-based questionnaire study of childhood, adolescent, and young adult cancer survivors. Using the NOR-CAYACS data, we aimed to:

1. Describe the occurrence of self-reported late effects, stratified by diagnosis and treatment;
2. Describe the survivors’ self-report of follow-up care for late effects received, and

In survivors with at least one reported late effect:

3. Identify factors associated with follow-up care for late effects received

## Patients and methods

### The NOR-CAYACS study

Participants eligible for the Norwegian CAYACS study (NOR-CAYACS) were identified through the CRN [32]. We included survivors who were diagnosed between 1985 and 2009, with any childhood cancer (CC, excluding central nervous system tumors) at ages 0–18 years or at the age 19–39 years with one of the following diagnosis: breast cancer (BC, ICD-10: C50, stage ≤ III), colorectal cancer (CRC, ICD-10: C18–20), non-Hodgkin lymphoma (NHL, ICD-10: C82–85), leukemias (LEUK, ICD-10: C91–96), and a random subsample of malignant melanomas (MM, ICD-10: 43). These diagnostic groups were chosen on the background of relatively good prognosis, expected risk of late effects, and lack of participation in other studies at our department. Other common AYA diagnostic groups, such as Hodgkin lymphoma and cervical and testicular cancer, were not included due to inclusion in other ongoing studies at our department, to avoid double publications and participation exhaustion. We excluded survivors who in the past 5 years preceding our study were diagnosed with a new cancer diagnosis as registered in the CRN, and/or those who self-reported relapse, and survivors self-reporting currently receiving oncological treatment. Eligible survivors were mailed an invitation to participate from the Oslo University Hospital including study information, an informed consent form, the questionnaire, and a pre-addressed pre-paid return envelope. No incentives to participate were offered. A reminder was sent by mail to survivors who had not responded after 5 months (more details about the NOR-CAYACS questionnaire are described in a previous paper [32]).

### Outcome variables from the questionnaire

#### Self-reported late effects

Participants were asked to report if they had ever experienced any of 17 different late effects: concentration and memory problems, fatigue, psychological reactions, hormonal changes, radiation damage, dental problems, numbness in hands/feet, reduced fertility, lymphedema, sexual problems, muscle cramps, neurological pain, hearing problems, lung problems, osteoporosis, new cancer, and heart disease. The list of late effects was constructed in two steps. First, we reviewed the literature and selected late effects that other studies had included in their questionnaires (e.g., [30, 31]). Second, a pediatric oncologist, two oncologists, and a psychiatrist with clinical and research expertise in late effects reviewed and adjusted the selected late effects (ER, SDF, CK, and JHL). The aim was to include similar late effects used in similar studies to allow for comparisons across studies and to capture survivors’ possible clinical complaints. As many survivors were unlikely to have received information about late effects from their clinicians given the long time since diagnosis, we provided a brief explanation of late effects in the questionnaire before the list of late effects and follow-up care. We created a cumulative late effects score by summarizing the number of all reported late effects and dividing them into four categories (no late effects, 1–2 late effects, 3–4 late effects, and 5 or more late effects).

#### Follow-up care for late effects

One question assessed whether the participants were currently receiving, or had received, specific follow-up care for late effects after cancer treatment (“Do you receive/have received care especially for late effects after cancer treatment?” Response alternatives: yes, no, don’t know). If yes, they were asked to indicate from whom they received the follow-up care: physician at treating hospital, other specialist physician, general practitioner (GP), or community cancer nurse.

### Exposure variables

#### Exposure variables from the CRN

Basic clinical and demographic information was provided by the CRN, including age, gender, diagnostic group, and age at diagnosis. Time since first diagnosis was calculated as years from cancer diagnosis until data extraction from the CRN in May 2015. Age at survey was calculated as age in years in May 2015.

#### Exposure variables from the questionnaire

*Treatment* was self-reported by the survivors and categorized into 4 groups: minimal (melanoma survivors with minimal surgery only), local (local surgery and/or radiotherapy), systemic (single chemotherapy or other systemic treatments), or multimodal (systemic treatment with surgery, radiotherapy, or stem cell transplantation) treatment [33]. *Relapse*: survivors indicated whether they had experienced a relapse or not (yes, no). *Education* was dichotomized into the following: ≤ high school which includes all schooling up to and including high school (13 years of schooling) and > high school includes any higher education at university or university college level.

### Statistics

We used descriptive statistics with numbers and proportions or means with standard deviations (SD) to present the characteristics of the participating survivors. To compare responders with non-responders, univariable logistic regression models with response status as outcome were used.

For aim 1, we used numbers and proportions to describe self-reported late effects overall and by cancer diagnosis and treatment. Chi-squared statistics was used to compare the late effects across the cancer types. For aim 2, we used numbers and proportions to describe follow-up care, stratified by cancer diagnosis, treatment, and number of late effects. For aim 3, we used univariable and multivariable logistic regression models with having received/receiving follow-up care as outcome and clinical and sociodemographic factors as exposure variables. For this analysis, we only included survivors who reported at least one late effect. Factors associated with the outcome in the univariable model with a *p* value < 0.05 were entered in the multivariable model. Gender and age at survey were kept a priori in the multivariable model. Because of multicollinearity between diagnosis and treatment, only treatment was included in the final model. A *p* value < 0.05 was considered significant and all tests were two sided. All analyses were performed in SPSS (IBM SPSS Statistics 24).

### Ethical considerations

The study was granted concession by The Norwegian Data Protection Authority (15/00395-2/CGN) and approved by the Regional Committee for Medical Research Ethics (2015/232 REK sør-øst B), and the Data Protection Officer at Oslo University Hospital and the Norwegian Cancer Registry.

## Results

### Patient characteristics

**Fig. 1:**
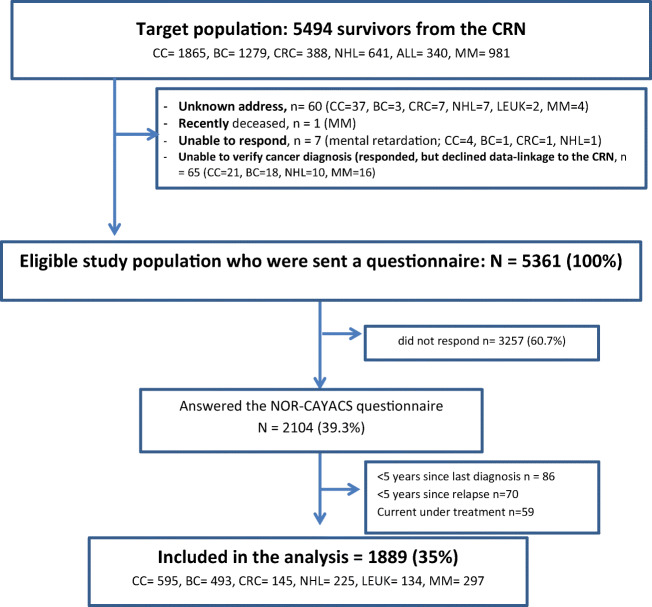
Flow diagram from target population of survivors in the Cancer Registry of Norway to the sample included in the analysis. CC childhood cancer, CRC colorectal cancer, NHL non-Hodgkin lymphoma, LEUK leukemia, MM malignant melanomas

Of the 5361 survivors invited, 2104 (39%) responded and 1889 (35%) were included in the current study (Fig. 1). In brief, responders were more likely to be females, slightly older at diagnosis and survey, longer time since diagnosis, and be diagnosed with BC, CRC, NHL, or LEUK than non-responders (data provided in Supplementary Table 1). This is in line with the non-response bias analysis done previously in a larger sample from the NOR-CAYACS study [32].

The sample consisted of 31% CC, 26% BC, 8% CRC, 12% NHL, 7% LEUK, and 16% MM survivors (Table 1). Two-thirds of the participants were female (68%) and the majority (57%) had higher education. Multimodal treatments were received by 58% of the survivors. Mean age at survey was 30 years among the CC and 49 years among the other diagnostic groups, diagnosed on average at the age of 11 and 33 years, with a mean of 19 and 15 years since diagnosis, respectively. Nine percent (*n* = 171) of the survivors had experienced a relapse before 2010 (5 years prior to study inclusion).

**Table 1:** Characteristics of participants included in the study (*N* = 1889)

	Total (*N* = 1889)	CC (*n* = 595)	BC (*n* = 493)	CRC (*n* = 145)	NHL (*n* = 225)	LEUK (*n* = 134)	MM (*n* = 297)
Sex							
Female	1281 (67.8%)	338 (56.8%)	493 (100.0%)	75 (51.7%)	104 (46.2%)	61 (45.5%)	210 (70.7%)
Male	608 (32.2%)	257 (43.2%)	0 (0.0%)	70 (48.3%)	121 (53.8%)	73 (54.5%)	87 (29.3%)
Education^a^							
< High school	812 (43.0%)	270 (45.4%)	209 (42.4%)	57 (39.3%)	96 (42.7%)	62 (46.3%)	118 (39.7%)
> High school	1077 (57.0%)	325 (54.6%)	284 (57.6%)	88 (60.7%)	129 (57.3%)	72 (53.7%)	179 (60.3%)
Treatment^b^							
Minimal	279 (14.8%)	0 (0.0%)	0 (0.0%)	0 (0.0%)	0 (0.0%)	1 (0.4%)	278 (93.6%)
Local treatment	204 (10.8%)	70 (11.8%)	39 (7.9%)	84 (57.9%)	11 (4.9%)	0 (0.0%)	0 (0.0%)
Systemic only	301 (16.0%)	162 (27.2%)	3 (0.7%)	2 (1.4%)	46 (20.5%)	78 (58.2%)	10 (3.4%)
Multimodal treatments	1102 (58.4%)	363 (61.0%)	450 (91.5%)	59 (40.7%)	166 (74.1%)	55 (41.0%)	9 (3.0%)
Relapse	221 (11.8%)	80 (13.5%)	50 (10.2%)	12 (8.3%)	39 (17.5%)	22 (16.7%)	18 (6.1%)
	Mean (SD, range)	Mean (SD, range)	Mean (SD, range)	Mean (SD, range)	Mean (SD, range)	Mean (SD, range)	Mean (SD, range)
Age at survey	43.2 (11.8, 18–64)	30.2 (7.8, 18–48)	49.8 (6.9, 30–64)	49.0 (9.0, 27–64)	48.5 (8.2, 26–64)	46.6 (8.3, 27–63)	49.7 (8.1, 29–64)
Age at diagnosis	25.8 (11.8, 0–39)	10.5 (6.0, 0–18)	35.3 (3.5, 21–39)	33.8 (4.9, 20–39)	30.8 (5.6, 19–39)	29.1 (5.9, 19–39)	31.5 (5.7, 19–39)
Time since first diagnosis	16.8 (6.9, 5–30)	19.2 (6.6, 5–30)	13.9 (5.9, 5–30)	14.6 (7.4, 5–30)	17.2 (6.9, 5–30)	16.9 (6.2, 5–29)	17.6 (6.9, 5–30)

*CC* childhood cancer, *BC* breast cancer, *CRC* colorectal cancer, *NHL* non-Hodgkin lymphoma, *LEUK* leukemias, *MM* malignant melanomas

^a^“< High school” includes all schooling up to and including high school (13 years of schooling); “> High school” includes any higher education at university or university college level. ^b^Missing treatment information on 3 patients. Minimal (melanoma survivors with minimal surgery only), local (local surgery and/or radiotherapy), systemic (single chemotherapy or other systemic treatments), or multimodal (systemic treatment with surgery, radiotherapy, or stem cell transplantation) treatment.

### Self-reported late effects

**Fig. 2:**
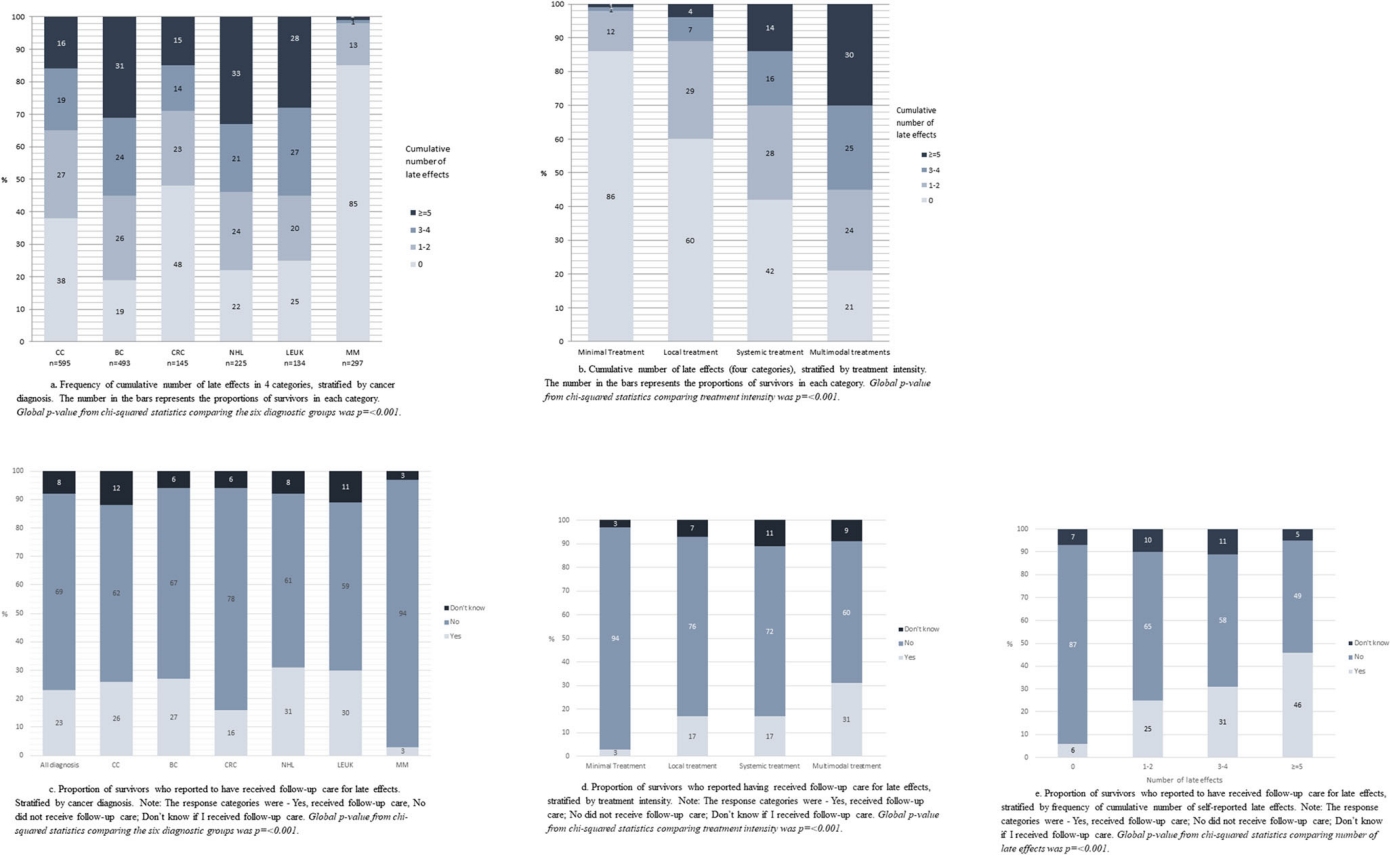
**a** Frequency of cumulative number of late effects in 4 categories, stratified by cancer diagnosis. The number in the bars represents the proportions of survivors in each category. Global *p* value from chi-squared statistics comparing the six diagnostic groups was *p* = < 0.001. **b** Cumulative number of late effects (four categories), stratified by treatment intensity. The number in the bars represents the proportions of survivors in each category. Global *p* value from chi-squared statistics comparing treatment intensity was *p* = < 0.001. **c** Proportion of survivors who reported to have received follow-up care for late effects. Stratified by cancer diagnosis. The response categories were as follows: Yes, received follow-up care; No, did not receive follow-up care; Don’t know if I received follow-up care. Global *p* value from chi-squared statistics comparing the six diagnostic groups was *p* = < 0.001. **d** Proportion of survivors who reported having received follow-up care for late effects, stratified by treatment intensity. The response categories were as follows: Yes, received follow-up care; No, did not receive follow-up care; Don’t know if I received follow-up care. Global *p* value from chi-squared statistics comparing treatment intensity was *p* = < 0.001. **e** Proportion of survivors who reported to have received follow-up care for late effects, stratified by frequency cumulative number of self-reported late effects. The response categories were as follows: Yes, received follow-up care; No, did not receive follow-up care; Don’t know if I received follow-up care. Global *p* value from chi-squared statistics comparing number of lose effects was *p* = < 0.001

A wide range of late effects were reported, with 61.5% reporting at least one late effect (median 2.4, 0–14) (Table 2). The most commonly reported late effects were concentration and memory problems (28.1%), fatigue (25.2%), psychological reactions (22.2%), and hormonal changes (20.5%), but they differed by cancer type (Table 2, Fig. 2a; *p* = < 0.001). When categorized, 23% reported 1–2 late effects, 18% 3–4 late effects, and 20% reported 5 or more late effects. Among the MM survivors, 15% reported at least one late effect compared with 81% of BC survivors (*p* = < 0.001) (Fig. 2a). Survivors after BC and NHL reported the highest number of late effects, of which 30% BC and 33% NHL reported 5 or more late effects.

**Table 2:** Frequencies of individual and cumulative numbers of self-reported late effects, overall and by cancer diagnosis—ordered by frequency in the total sample

Late effect	Total (*N* = 1889)	CC (*n* = 595)	BC (*n* = 493)	CRC (*n* = 145)	NHL (*n* = 225)	LEUK (*n* = 134)	MM (*n* = 297)	*p* value*
	*N* (%)	*n* (%)	*n* (%)	*n* (%)	*n* (%)	*n* (%)	*n* (%)	
Concentration and memory problems	531 (28.1)	170 (28.6%)	201 (40.8%)	34 (23.4%)	75 (33.3%)	47 (35.1%)	4 (1.3%)	< 0.001
Fatigue	476 (25.2)	133 (22.4%)	174 (35.3%)	34 (23.4%)	88 (39.1%)	43 (32.1%)	4 (1.3%)	< 0.001
Psychological reactions	420 (22.2)	138 (23.2%)	139 (28.2%)	26 (17.9%)	59 (26.2%)	30 (22.4%)	28 (9.4%)	0.084
Hormonal changes	388 (20.5)	95 (16.0%)	185 (37.5%)	18 (12.4%)	55 (24.4%)	33 (24.6%)	2 (0.7%)	< 0.001
Radiation damage	316 (16.7)	75 (12.6%)	175 (35.5%)	13 (9.0%)	48 (21.3%)	3 (2.2%)	2 (0.7%)	< 0.001
Dental problems	323 (17.1)	131 (22.0%)	67 (13.6%)	13 (9.0%)	65 (28.9%)	46 (34.3%)	1 (0.3%)	< 0.001
Numbness in hands/feet	304 (16.1)	87 (14.6%)	80 (16.2%)	23 (15.9%)	78 (34.7%)	35 (26.1%)	1 (0.3%)	< 0.001
Reduced fertility	300 (15.9)	98 (16.5%)	63 (12.8%)	22 (15.2%)	69 (30.7%)	46 (34.3%)	2 (0.7%)	< 0.001
Lymphedema	277 (14.7)	23 (3.9%)	191 (38.7%)	8 (5.5%)	31 (13.8%)	15 (11.2%)	9 (3.0%)	< 0.001
Sexual problems	263 (13.9)	43 (7.2%)	120 (24.3%)	26 (17.9%)	39 (17.3%)	33 (24.6%)	2 (0.7%)	< 0.001
Muscle cramps	231 (12.2)	75 (12.6%)	56 (11.4%)	8 (5.5%)	55 (24.4%)	35 (26.1%)	2 (0.7%)	< 0.001
Neurological pain	227 (12.0)	58 (9.7%)	76 (15.4%)	18 (12.4%)	41 (18.2%)	27 (20.1%)	7 (2.4%)	< 0.001
Hearing problem	137 (7.3)	58 (9.7%)	37 (7.5%)	8 (5.5%)	15 (6.7%)	18 (13.4%)	1 (0.3%)	0.072
Lung disease	106 (5.6)	49 (8.2%)	15 (3.0%)	0 (0.0%)	28 (12.4%)	14 (10.4%)	0 (0.0%)	< 0.001
Osteoporosis	93 (4.9)	20 (3.4%)	44 (8.9%)	4 (2.8%)	11 (4.9%)	13 (9.7%)	1 (0.3%)	< 0.001
Heart disease	83 (4.4)	38 (6.4%)	11 (2.2%)	1 (0.7%)	25 (11.1%)	8 (6.0%)	0 (0.0%)	< 0.001
New cancer	52 (2.8)	16 (2.7%)	14 (2.8%)	2 (1.4%)	9 (4.0%)	8 (6.0%)	3 (1.0%)	0.185
Cumulative number of late effects								< 0.001
0	727 (38.5)	226 (38.0%)	94 (19.1%)	70 (48.3%)	49 (21.8%)	34 (25.4%)	254 (85.5%)	
1–2	437 (23.1)	157 (26.4%)	129 (26.2%)	33 (22.8%)	55 (24.4%)	26 (19.4%)	37 (12.5%)	
3–4	339 (17.9)	115 (19.3%)	118 (23.9%)	20 (13.8%)	46 (20.4%)	36 (26.9%)	4 (1.3%)	
≥ = 5	386 (20.5)	97 (16.3%)	152 (30.8%)	22 (15.2%)	75 (33.3%)	38 (28.4%)	2 (0.7%)	

*CC* childhood cancer, *BC* breast cancer, *CRC* colorectal cancer, *NHL* non-Hodgkin lymphoma, *LEUK* leukemias, *MM* malignant melanomas. *Global *p* value from chi-squared statistics comparing the five diagnostic groups, excluding malignant melanomas

Among the survivors who had received multimodal treatments, 55% reported 3 or more late effects compared with 30% for systemic treatment, 11% for local treatment, and 2% for minimal surgery (Fig. 2b; *p* = < 0.001).

### Follow-up care for late effects

About two-thirds of all survivors (69%) reported not having received/not receiving follow-up care for late effects, while 23% had received/were receiving such care and 8% were unsure (Fig. 2c). These proportions differed across diagnosis (*p* = < 0.001). Lowest proportions of receiving follow-up care were reported among the MM (3%) and CRC group (16%) and highest among the CC, BC, LEUK, and NHL group (26–31%).

Treatment intensity was positively associated with receiving follow-up care. Compared with the other groups, survivors treated with multimodal treatments reported the highest proportions of receiving or having received follow-up care for late effects (Fig. 2d; *p* = < 0.001). Similarly, increasing number of experienced late effects was associated with increasing proportion of receiving or having received follow-up care (Fig. 2e; *p* = < 0.001).

The follow-up care for late effects was most often provided by hospital doctor (64%) (physician at treating hospital (28%), other medical specialists (36%)), general practitioner (44%), and community cancer nurse (0.2%).

#### Factors associated with follow-up care

In the univariable regression models, age at survey, higher education, diagnosis, treatment, and increasing number of late effects were associated with having received/receiving follow-up care (all *p* < 0.05; Table 3). In the multivariable model, higher likelihood of receiving/having received follow-up care for late effects was associated with lower age at survey compared with older age (OR 0.98 (CI 0.97–0.99)), higher education compared with those with low education (OR 1.34 (CI 1.08–1.80); *p* = 0.012), and reporting more than two late effects (3 or 4: OR 1.33 (CI 0.96–1.85); 5 or more: OR 2.54 (CI 1.85–3.48), *p* = < 0.001).

**Table 3:** Factors associated with receiving follow-up care for late effects in survivors reporting at least one late effect. Univariable and multivariable logistic regression models are presented (*n* = 1162)

	Factors associated with receiving follow-up care^a^				
	Univariable			Multivariable	
	Mean	OR (95% CI)	*p* value		*p* value
Age at diagnosis	26.3	0.99 (0.98–1.00)	0.113		
Age at survey	42.8	0.99 (0.98–0.99)	0.016	0.98 (0.97–0.99)	0.001
Time since first diagnosis	15.9	0.99 (0.97–1.01)	0.204		
	*n*	OR (95% CI)	*p* value	OR (95% CI)	*p* value
Sex			0.380		0.688
Female	828	1			
Male	334	0.89 (0.67–1.16)		0.94 (0.71–1.26)	
Education^b^			0.011		0.012
≤ High school	503	1		1	
> Higher education	659	1.38 (1.08–1.84)		1.39 (1.08–1.80)	
Diagnosis			0.028		
Childhood cancer	368	1			
Melanoma	43	0.44 (0.21–0.95)			
Breast cancer	398	0.77 (0.57–1.04)			
Colorectal cancer	75	0.42 (0.23–0.77)			
Non-Hodgkin’s lymphoma	175	0.84 (0.58–1.23)			
Leukemias	99	0.98 (0.62–1.55)			
Treatment^c^			0.006		0.124
Minimal	38	1		1	
Local	81	1.76 (0.68–4.55)		1.48 (0.57–3.89)	
Systemic	174	1.45 (0.60–3.54)		0.91 (0.36–2.29)	
Multimodal	869	2.47 (1.07–5.47)		1.43 (0.61–3.38)	
Relapse			0.071		
Yes	171	1.36 (0.97–1.90)			
No	987	1			
Number of late effects			< 0.001		< 0.001
1–2	437	1		1	
3–4	339	1.35 (0.98–1.85)		1.33 (0.96–1.85)	
> = 5	386	2.53 (1.88–3.40)		2.54 (1.85–3.48)	

*CI* confidence interval, *OR* odd ratio

^a^Outcome is codes as 0 = not receiving follow-up care and 1 = having received/receiving follow-up care. Because of multicollinearity between diagnosis and treatment, only treatment was included in the final model. ^b^ “< High school” includes all schooling up to and including high school (13 years of schooling); “> High school” includes any higher education at university of university college level. ^c^Missing treatment information on 3 patients. Minimal (melanoma survivors with minimal surgery only), local (local surgery and/or radiotherapy), systemic (single chemotherapy or other systemic treatments), or multimodal (systemic treatment with surgery, radiotherapy or stem cell transplantation) treatment

## Discussion

In this study, we present information on self-reported late effects among 1889 long-term CAYACS and their experience with follow-up care for these. More than 60% of the CAYACS reported at least one late effect. Survivors of BC, NHL, and LEUK diagnosed between the ages of 19 and 39 years reported the highest number of late effects. The majority of the survivors reported not having received or not receiving follow-up for late effects. Lower age, higher number of experienced late effects, and a higher level of education were associated with higher probability of receiving follow-up care for late effects.

The most commonly reported late effects were concentration and memory problems, fatigue, psychological reactions, and hormonal changes, which is in line with studies of childhood cancer survivors [1–3], but has rarely been studied in survivors of CRC and MM. Although not life-threatening, these are problems that can have significant impact on quality of life and participation in activities of daily living including work life issues [5]. This is in line with a recent study by our group based on the same cohort, where higher risks of unemployment and low work ability were significantly associated with both self-reported treatment intensity and higher number of late effects [34].

Of the survivors reporting one late effect or more, only one-quarter reported to receive/having received follow-up care specifically for late effects. Survivors of NHL, BC, LEUK, and CC reported the highest proportion of received care (26–31%) compared with 16% of CRC and 3% among MM survivors. The relatively low number of survivors attending follow-up care corresponds to reports on childhood cancer survivors from other countries [24–26, 35]. This is also in line with previous studies from our research group among other groups of cancer survivors mostly diagnosed at young ages [36, 37].

The low number of survivors reporting to receive/having received follow-up for late effects could potentially be related to the nature of the late effects the survivors reported. Memory and concentration problems, fatigue, and psychological reactions are perhaps less commonly assessed, monitored, and treated in follow-up care than somatic late effects, e.g., hormonal problems, heart problems, or secondary cancers [24]. These results support previous literature on long-term follow-up care calling for increased attention to psychological and social late effects in addition to the physical consequences of cancer [33, 38].

Low rates of reported follow-up care could also reflect the structure of the Norwegian healthcare system. Long-term survivors are generally not in formal follow-up care, but are expected to consult their general practitioner for health problems as a first port-of-call. However, survivors’ lack of knowledge of late effects may delay their recognition of developing problems and may delay seeking necessary health care [39].

In multivariate analysis, lower age at survey, a higher number of late effects, and higher education were the significant predictors of receiving/having received follow-up care. While high cost of care and lack of insurance have been cited as potential barriers of follow-up care among CAYACS elsewhere [40], all Norwegian cancer survivors have access to healthcare given the publicly funded healthcare system. Once diagnosed, late effects are followed up by either general practitioners and/or relevant medical specialists. The finding that younger age is associated with greater likelihood of receiving follow-up care is in line with earlier findings [40]. One reason for this finding in the present study may be that the CC group is both more likely to receive follow-up care and has a lower mean age than the other diagnosis groups. That higher education is associated with improved follow-up care suggests that, although health care is available to all, its accessibility may be moderated by characteristics of the survivor such as socioeconomic status [41] and health literacy [42]. This is in line with previous research showing that survivors with higher socio-economic status, related to higher level of education, reported to receive follow-up care more frequently than survivors with lower education socioeconomic status in similar healthcare system [25, 26].

That more than half of the survivors reporting three or more late effects reported not to receive/have received follow-up for these calls for a more structured system for cancer survivorship care. To address the gap between the primary and specialized healthcare system in taking care for young cancer survivors, several models have been proposed, based on shared care between the treating hospital and the general practitioner, balancing the individual survivors’ existing late effect and risk for future morbidity [13, 43]. Except for early detection of new cancer activity, the goals for cancer survivorship programs include detection, early treatment, and prevention of late effects. In a country like Norway, a model principally based on cooperation between the cancer survivor and the primary health care is probably adequate for most cancer survivors if supported by medical specialists when needed. However, for the cancer survivors with the most complex and highest risks for serious late effects, there is a need for care pathways that link specialty and primary healthcare system together [39, 43].

### Strengths and limitations

A major strength of our study is the unselected, large nationwide, population-based cohort of long-term survivors representing a range of diagnostic groups. The high quality of the CRN data and the use of unique national identifying numbers in Norway allowed us to track and contact the whole population of eligible survivors, including those who may have been lost and disengaged from care. As such, these data provide a unique “snap-shot” of generally hard to reach populations.

There are several limitations. First, the results are based on self-report by the survivors and do not always reflect objectively verified late effects. Even though the wording of the question specified the conditions as late effects after cancer treatment, there is a risk that health problems not directly related to the cancer trajectory might have been attributed as late effects. However, other studies on the occurrence of late effects among childhood cancer survivors have also been based on self-report [2, 7]. Second, although our modest response rate might rise concerns of non-response bias risk, we found limited evidence of such in the same cohort [32]. There might be a concern that survivors who experience late effects are more inclined to answer the questionnaire which could lead to an overestimation of the prevalence of late effects. Based on the demographic and clinical information provided by the CRN for the whole population, we found no risk for an overrepresentation of survivors with fewer or more late effects than the non-responders in the sample using an inverse probability weighted model of participation [32]. Third, the cross-sectional design does not allow for causal relationships to be investigated. Fourth, using number of late effects as a proxy for the overall burden of late effects is a crude measure because the late effects are not necessarily mutually exclusive, and they were not weighted for severity. However, to construct a list of mutually exclusive late effects which can be subjected to appropriate severity weighting requires a level of details and length of questionnaire beyond the scope of the NOR-CAYACS study. Although crude, this proxy measure of late effect burden correlates well with treatment intensity, a well-known risk factor for late effects, adding to its face validity. Fifth, we only assessed “follow-up care specifically for late effects” in addition to routine follow-up care in one question. The distinction regarding these types of care was explained in the questionnaire. As the aim was to assess how many had received such extra care, distinguishing between such care received in the past and care currently received was considered not to be essential, albeit represents a minor limitation of the study. Last, our numbers for new cancers as late effects may be under-reported given survivors with any new diagnoses of cancers in the last 5 years prior to the study were excluded.

## Conclusion

In conclusion, in this large, unselected sample of long-term CAYACSs, the majority reported having received multimodal cancer treatment, placing them at risk of developing late effects. Two-thirds reported experiencing at least one late effect, but the majority of these reported not receiving or having received follow-up care for late effects. This suggests that formal models of long-term follow-up care should be developed which ensure equal access to long-term follow-up care for all cancer survivors.

### Implications for cancer survivors

There is currently a gap between survivors’ experience of long-term follow-up care as recommended in international literature and guidelines. Survivors with low educational levels seem to be at higher risk of not receiving adequate follow-up care compared with survivors with higher education. Our data indicate that long-term CAYACS at risk of developing late effects would benefit from a formal model of follow-up care to ensure they receive recommended follow-up care.

## Electronic supplementary material

ESM 1 (DOCX 25 kb)

## Data Availability

Not available. The data will not be deposited.
