# Supplementary material for: Self-reported late effects and long-term follow-up care among 1889 long-term Norwegian Childhood, Adolescent, and Young Adult Cancer Survivors (the NOR-CAYACS study)
Source: Support Care Cancer. 2020 Oct 3;29(6):2947–57. doi: 10.1007/s00520-020-05790-6 (PMC8062364; doi:10.1007/s00520-020-05790-6)
Supplement: Supplementary file 1 — (DOCX 25 kb) [file 520_2020_5790_MOESM1_ESM.docx]

**Supplementary Table 1.** Characteristics of responders and non-responders based on information from the CRN.

|  | Responders | Non-responders |  |
| --- | --- | --- | --- |
|  | Mean /Median (SD, range) | Mean/median (SD, range) | *p^1^* |
| **n** | 1889 | 3257 |  |
| **Age at survey (years)** | 43.6/45.3 (11.8, 18-65) | 41.0/43.3 (12.0, 18-64) | <0.001 |
| **Time since first diagnosis (years)** | 16.8/17.0 (6.9, 5-30) | 15.8/15.0 (6.7, 5-30) | <0.002 |
| **Age at diagnosis (years)** | 25.8/30.0 (11.7, 0-39) | 24.7/28.0 (12.1, 0-39) | <0.001 |
|  | n (%)^2^ | n (%)^2^ | *p* |
| **Gender** |  |  | <0.001 |
| Female | 1281 (67.8) | 1923 (59.0) |  |
| Male | 608 (32.2) | 1334 (41.0) |  |
| **Diagnostic group^3^** |  |  | <0.001 |
| CC | 595 (31.5) | 1187 (36.4) |  |
| BC | 493 (26.1) | 660 (20.3) |  |
| CRC | 145 (7.7) | 225 (6.9) |  |
| NHL | 225 (11.9) | 370 (11.4) |  |
| LEUK | 134 (7.1) | 181 (5.6) |  |
| MM | 297 (15.7) | 634 (19.5) |  |

Notes:

^1^P-values derived from univariable logistic regression model.

^2^Column percentages are reported

^3^The diagnosis of non-Hodgkin lymphoma and LEUK may occur in both the CC and AYA diagnostic groups of NHL and LEUK respectively depending on whether they were diagnosed before or after the age of 19 years. CC=childhood cancer, BC = breast cancer, CRC = Colorectal cancer, NHL = non-Hodgkin lymphoma, LEUK = Leukemias, MM = Malignant melanoma.
